# Species status of *Neisseria gonorrhoeae*: evolutionary and epidemiological inferences from multilocus sequence typing

**DOI:** 10.1186/1741-7007-5-35

**Published:** 2007-09-07

**Authors:** Julia S Bennett, Keith A Jolley, P Frederick Sparling, Nigel J Saunders, C Anthony Hart, Ian M Feavers, Martin CJ Maiden

**Affiliations:** 1 The Peter Medawar Building for Pathogen Research and Department of Zoology, University of Oxford, South Parks Road, Oxford, OX1 3SY, UK; 2 Department of Microbiology and Immunology, School of Medicine, University of North Carolina at Chapel Hill, Chapel Hill, North Carolina, USA; 3 The Sir William Dunn School of Pathology, University of Oxford, South Parks Road, Oxford, OX1 3RE, UK; 4 Department of Medical Microbiology and Genitourinary Medicine, Royal Liverpool University Hospital, Duncan Building, Daulby Street, Liverpool L69 3GA, UK; 5 Division of Bacteriology, National Institute for Biological Standards and Control, South Mimms, Potters Bar, Hertfordshire, EN6 3QG, UK

## Abstract

**Background:**

Various typing methods have been developed for *Neisseria gonorrhoeae*, but none provide the combination of discrimination, reproducibility, portability, and genetic inference that allows the analysis of all aspects of the epidemiology of this pathogen from a single data set. Multilocus sequence typing (MLST) has been used successfully to characterize the related organisms *Neisseria meningitidis *and *Neisseria lactamica*. Here, the same seven locus *Neisseria *scheme was used to characterize a diverse collection of *N. gonorrhoeae *isolates to investigate whether this method would allow differentiation among isolates, and to distinguish these three species.

**Results:**

A total of 149 gonococcal isolates were typed and submitted to the *Neisseria *MLST database. Although relatively few (27) polymorphisms were detected among the seven MLST loci, a total of 66 unique allele combinations (sequence types, STs), were observed, a number comparable to that seen among isolate collections of the more diverse meningococcus. Patterns of genetic variation were consistent with high levels of recombination generating this diversity. There was no evidence for geographical structuring among the isolates examined, with isolates collected in Liverpool, UK, showing levels of diversity similar to a global collection of isolates. There was, however, evidence that populations of *N. meningitidis*, *N. gonorrhoeae *and *N. lactamica *were distinct, with little support for frequent genetic recombination among these species, with the sequences from the *gdh *locus alone grouping the species into distinct clusters.

**Conclusion:**

The seven loci *Neisseria *MLST scheme was readily adapted to *N. gonorrhoeae *isolates, providing a highly discriminatory typing method. In addition, these data permitted phylogenetic and population genetic inferences to be made, including direct comparisons with *N. meningitidis *and *N. lactamica*. Examination of these data demonstrated that alleles were rarely shared among the three species. Analysis of variation at a single locus, *gdh*, provided a rapid means of identifying misclassified isolates and determining whether mixed cultures were present.

## Background

Gonorrhoea, caused by the bacterium *Neisseria gonorrhoeae*, remains one of the most common sexually transmitted diseases contributing a substantial burden of morbidity, mortality and infertility worldwide. The disease is treatable and curable, but no vaccine is available. Consequently the control of this important disease depends on the identification and treatment of infected individuals and their contacts in transmission networks. High-resolution and reproducible typing methods for clinical isolates of the gonococcus are therefore central to the control of gonococcal infection. Knowledge of the gonococcal strains circulating both locally and globally, and of temporal changes in the prevalence of these strains, would identify transmission patterns and may assist in prevention and control of this disease.

Many typing schemes have been developed for *N. gonorrhoeae *but no single typing scheme has been generally adopted, and the lack of a single, generally accepted typing method has impeded the sharing of epidemiological data between laboratories. Auxotyping and serotyping are often applied to gonococci and these techniques are frequently combined, but they do not always provide sufficient resolution to distinguish between epidemiologically related and unrelated isolates [1].

Molecular based typing schemes [2-6] provide better discrimination among isolates. One method, multilocus enzyme electrophoresis (MLEE), which indexes variation in housekeeping genes, has been utilized to characterize gonococci, and has shown that strains with an AHU- (arginine, hypoxanthine and uracil requiring) auxotype are uniform, despite frequent recombination among gonococci [7]. AHU- isolates have been linked to disseminated gonococcal infection (DGI) [8], which is related to the penicillin sensitive phenotype usually found among these isolates [9].

Methods that use nucleotide sequencing, however, [10-13], are more portable, have greater definition, and make data storage in globally accessible databases via the internet easier. One method, based on the nucleotide sequence fragments from two gonococcal antigen genes under diversifying immune selection: *por *and *tbpB *(*N. gonorrhoeae *multi antigen sequence typing, NG-MAST) [14,15], provides a high level of discrimination. However the NG-MAST database only includes genotypes, consisting of two number allelic profiles and the nucleotide sequences, with no isolate data available.

One established method for the characterization of bacteria is multilocus sequence typing (MLST), a development of MLEE, and a highly discriminatory system for indexing the relatedness among isolates based on genetic variation present in genes under stabilising selection for conservation of metabolic function [16]. It is employed for the characterisation of many bacterial species, including the closely related pathogen *Neisseria meningitidis *and the commensal *Neisseria lactamica *[16-21].

An intriguing feature of gonococcal biology is the very close relationship of this bacterium to *N. meningitidis *and *N. lactamica*, which also have an obligate association with humans, but inhabit the mucosal surface of the nasopharynx rather than the urogenital tract. Application of the same MLST scheme to *N. gonorrhoeae*, is therefore advantageous as it can be used to analyse genetic relationships among gonococcal isolates, as well as among the neisseriae [22]. Another advantage of MLST is its ability to discriminate among species, facilitating species identification and the detection of mixed bacterial cultures. This paper describes a *N. gonorrhoeae *typing scheme that exploits the existing globally accessible *Neisseria *MLST database [23,24], which provides publicly available isolate information as well as nucleotide sequence data.

## Results

### Diversity among alleles and sequence types

A total of 66 sequence types (STs) were identified among the 149 gonococcal isolates analysed. The number of alleles at each locus ranged from two at *aroE *to 10 at *gdh *(Table 1). Of the 66 unique STs, 35 STs were represented by single isolates, 29 STs were represented by two to six isolates, ST-1579 was represented by 10 isolates and ST-1595 was represented by 12 AHU- isolates. Another AHU- isolate was identified as ST-5688, which differed from the ST-1595 AHU- isolates by a single synonymous polymorphism in the *gdh *allele. There were eight STs among isolates collected from 10 cases of DGI. A larger study would be necessary to investigate any relationships between invasive isolates and ST.

**Table 1:** Genetic variation in *Neisseria *MLST alleles

		149 *N. gonorrhoeae *isolates			217 *N. meningitidis *isolates			103 *N. lactamica *isolates		
Locus	Size (bp)	No. of alleles (no./100 isolates)	No. (%) of polymorphic sites	*d*_*N*_*d*_S_*	No. of alleles (no./100 isolates)	No. (%) of polymorphic sites	*d*_*N*_/*d*_*S*_	No. of alleles (no./100 isolates)	No. (%) of polymorphic sites	*d*_*N*_/*d*_*S*_

*abcZ*	432	7 (5.1)	6 (1.4)	0.177	21 (9.6)	75 (17.4)	0.074	12 (11.7)	45 (10.4)	0.154
*adk*	465	3 (2.2)	3 (0.6)	0.583	19 (8.7)	25 (5.4)	0.011	18 (17.5)	43 (9.3)	0.014
*aroE*	489	2 (1.5)	1 (0.2)	0†	21 (9.6)	135 (27.6)	0.295	16 (15.5)	45 (9.2)	0.464
*fumC*	465	9 (6.6)	7 (1.5)	0.069	29 (13.3)	48 (10.3)	0.010	19 (18.5)	44 (9.5)	0.042
*gdh*	501	10 (6.7)	6 (1.2)	0.147	19 (8.7)	26 (5.2)	0.049	26 (25.2)	46 (9.2)	0.047
*pdhC*	480	3 (2.2)	2 (0.4)	0.328	25 (11.5)	83 (17.3)	0.068	11 (17.5)	15 (3.1)	0.024
*pgm*	450	3 (2.2)	2 (0.4)	0.298	25 (11.5)	81 (18.0)	0.113	22 (21.4)	96 (21.3)	0.095

*d_*N*_/*d*_*S *_the proportion of nonsynonymous to synonymous nucleotide substitutions

† No nonsynonymous changes present at this locus

**Figure 1:**
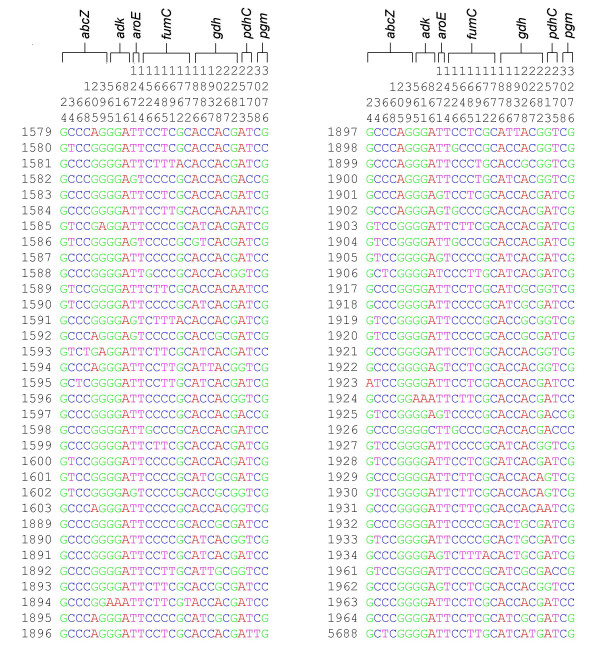
**Polymorphic sites in concatenated gonococcal housekeeping gene sequences**. The polymorphic sites are shown for each concatenated sequence of seven housekeeping gene fragments. The positions of the polymorphic sites, and the genes in which they occur are indicated along the top.

The allele sequences for each ST, concatenated in frame, were used to indicate the polymorphic sites within each ST, demonstrating the diversity present (Figure 1). Data for these isolates were submitted to the *Neisseria *MLST database [23,24] and were given ST designations and allele numbers in order of discovery, so that the first gonococcal ST identified in this study was designated ST-1579.

**Figure 2:**
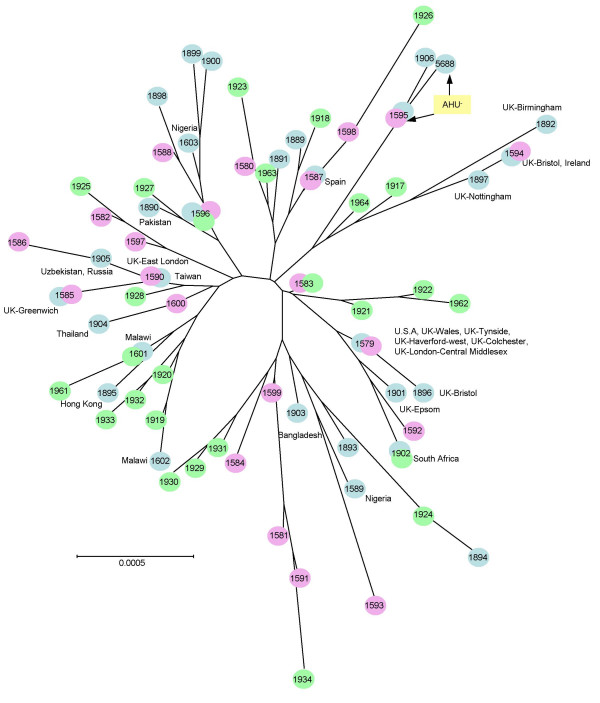
**The temporal and geographic distribution of 66 STs described for *N. gonorrhoeae***. A neighbour-joining tree was constructed from the concatenated MLST sequences from 66 STs obtained from 149 gonococcal isolates. Each circle denotes a particular ST and the countries and regions of isolation, if known are shown. Green circles indicate STs from isolates collected in Liverpool, UK between 1981 and 1991, pink circles indicate STs from isolates collected in Liverpool, UK between 2000 and 2001 and blue circles indicate STs from isolates collected elsewhere, including geographically and epidemiologically unlinked isolates. The two closely related STs obtained from the AHU- isolates are shown.

A neighbour-joining tree constructed from the concatenated allele sequences demonstrated the diversity of these isolates (Figure 2). Bacteria with the same STs were isolated in more than one location and some from more than one continent, while others demonstrated temporal persistence (Figure 2, Additional file 1).

### Comparisons of *N. gonorrhoeae *with *N. meningitidis *and *N. lactamica*

The allelic diversity within the 149 gonococcal isolates was compared with the diversity within 217 carried meningococci collected in the Czech Republic during 1993 [25] and a subset of 103 *N. lactamica *isolates collected as part of a longitudinal study of *N. lactamica *carriage in infants [20] (Table 1). The number of alleles and the percentage of polymorphic sites per allele were much greater for *N. meningitidis *and *N. lactamica *than for *N. gonorrhoeae*. The ratio of nonsynonymous to synonymous nucleotide substitutions (*d*_*N*_*/d*_*S*_), calculated as an average over the entire MLST fragment for each locus was < 1 for each species, evidence that the loci used in the *Neisseria *MLST scheme were not subject to diversifying selection.

The number of gonococcal STs was compared to the number of STs among the Czech meningococcal carriage collection, the *N. lactamica *collection, and the collection of 107 meningococcal isolates used to develop the first MLST scheme and chosen to represent the diversity of the meningococcal population worldwide [16] (Table 2). The number of STs per 100 isolates among the gonococci (44) was comparable to the numbers of STs per 100 isolates among the carried meningococci (41) and the collection of 107 meningococci (47). The collection of 103 *N. lactamica *isolates had the highest number of STs per 100 isolates (67). When the collection of gonococcal isolates was divided into individual datasets, the dataset of 53 gonococcal isolates collected worldwide revealed 57 STs per 100 isolates, and the 38 gonococcal isolates collected in Liverpool between 2000 and 2001 comprised 55 STs per 100 isolates, demonstrating a greater number of STs per 100 isolates than in either meningococcal isolate collection.

**Table 2:** Variability in *Neisseria *MLST STs

Datasets	No. of STs (estimated no. of STs/100 isolates)	No. (%) of isolates with unique STs
107 meningococcal isolates from the MLST reference strain database	50 (47)	40 (37)
217 carried meningococci collected in the Czech Republic during 1993	88 (41)	63 (29)
103 *N. lactamica *isolates, collected in Oxfordshire, UK	69 (67)	53 (51)
All 149 gonococcal isolates analysed	66 (44)	35 (23)
53 gonococcal isolates (collected worldwide)	30 (57)	20 (38)
58 gonococcal isolates collected in Liverpool between 1981–1989	26 (45)	13 (22)
38 gonococcal isolates collected in Liverpool between 2000–2001	21 (55)	11 (29)

In each of the collections analysed, many of the isolates had unique STs, with the percentage of unique STs among 53 gonococcal isolates collected worldwide (38%), comparable to that found among the collection of 107 meningococcal isolates (37%). The same percentage of unique alleles was found among 38 gonococcal isolates collected in Liverpool between 2000 and 2001, and the 217 Czech carried meningococci (29%).

Genetic divergence and gene flow (*F*_*ST*_) were calculated between 58 gonococcal isolates collected in Liverpool (1981–1989), 38 gonococcal isolates collected in Liverpool between 2000 and 2001, 53 gonococcal isolates collected worldwide, 217 Czech carried meningococci and 103 *N. lactamica *isolates (Table 3). Fixed differences were present between species but none among the three groups of gonococci. More polymorphisms were shared among the gonococcal groups than among the species, and the percentage nucleotide sequence divergence was greatest between species. The *N. lactamica *nucleotide sequences were the least similar to the gonococcal nucleotide sequences (9.46% divergence). The *F*_*ST *_value between the two gonococcal groups was close to zero (0.01, 0.02), whereas between gonococci and *N. lactamica *it was 0.79, and between gonococci and *N. meningitidis *it was 0.61. The three gonococcal isolate collections were not significantly different (*p *> 0.05), with no geographic or temporal structuring evident.

**Table 3:** Genetic divergence and gene flow between groups

	Fixed differences	Shared polymorphisms (total no. of polymorphisms)	Percentage mean nucleotide sequence divergence	*F*_ST_*
38 *N. gonorrhoeae *(Liverpool, 2000–2001)	0	15 (23)	0.14	0.01 (*p *> 0.05)
53 *N. gonorrhoeae *(collected worldwide)	0	15 (25)	0.15	0.02 (*p *> 0.05)
103 *N. lactamica *(Oxfordshire)	170	4 (530)	9.46	0.79 (*p *< 0.05)
217 *N. meningitidis *(Czech carriage)	64	3 (570)	6.91	0.61 (*p *< 0.05)

A total of 58 *N. gonorrhoeae *isolates collected in Liverpool between 1981 and 1989 were compared with collections of *N. gonorrhoeae, N. lactamica and N. meningitidis*.

*This statistic measures the extent of genetic differentiation and computes an average level of gene flow (*F*_*ST *_= 0 free genetic recombination, *F*_*ST *_= 1 recombination unlikely).

**Figure 3:**
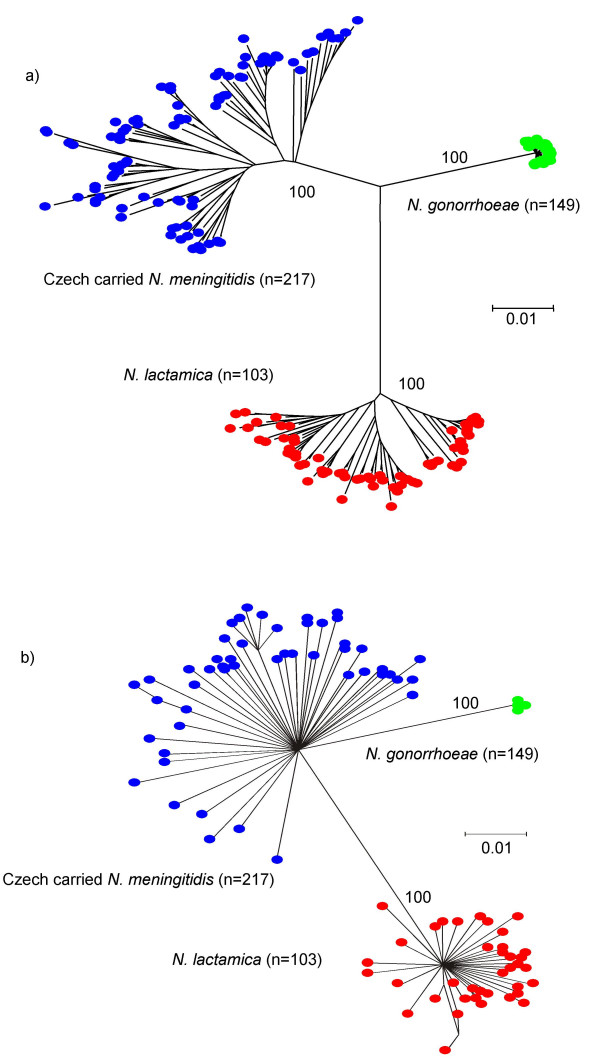
**MLST data resolves *N. gonorrhoeae*, *N. meningitidis *and *N. lactamica *into three clusters**. (a) A neighbour-joining tree was constructed from the concatenated MLST sequences for each species. (b) A splits graph was constructed from the concatenated MLST sequences for each species. Bootstrap values are indicated for the main branches (2000 replications).

To determine whether a clustering algorithm would delineate the three species, a neighbour-joining tree was constructed with the concatenated nucleotide sequences from the 149 *N. gonorrhoeae*, the 103 *N. lactamica*, and the 217 carried meningococci (Figure 3a). This showed three distinct clusters, corresponding to each of the three species, supported by bootstrap values of 100%. Bootstrap values within the clusters were very variable (not shown), suggesting relationships were not well resolved, a finding consistent with high levels of within species recombination. Similar clustering was shown using split decomposition analysis (Figure 3b) [26], although with this method, *N. gonorrhoeae *appeared to form a distinct cluster within the diversity of the meningococcus. No alleles were common to more than one species when 149 gonococci, 324 meningococci, and 103 *N*. *lactamica *isolates were analysed.

**Figure 4:**
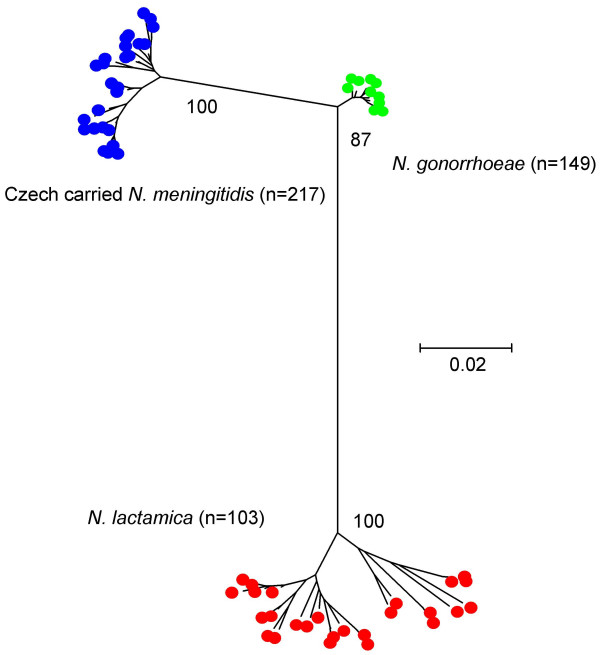
**Alleles from a single locus (*gdh*) resolved species specific clusters**. A neighbour-joining tree was constructed from the *gdh *allele sequences for each species. Bootstrap values are indicated for the main branches (2000 replications).

The alleles that make up the allelic profile of each ST were examined individually using neighbour-joining trees (data only shown for *gdh*). The tree for the alleles at the *gdh *locus resolved the species into three well-supported groups, producing a tree congruent with that obtained from the concatenated nucleotide sequences (Figure 4). The trees drawn from alleles at the other six loci did not resolve the three species into groups that were congruent with the concatenated nucleotide sequences, although the majority of alleles from the same species formed clusters, with the gonococcal alleles forming single tight groups for all seven loci.

## Discussion

It has been suggested that MLST of the pathogen *N. gonorrhoeae *would not provide sufficient discrimination between strains [27], due to the uniformity of its housekeeping genes [28]. The present study has shown that *N. gonorrhoeae *can be typed effectively using the same MLST scheme employed to characterize *N. meningitidis *[16] and *N. lactamica *[20], with a genotypic diversity comparable to that found among meningococcal isolates [25]. Despite high levels of horizontal genetic exchange among gonococci [2], MLST is robust because it is based on data from seven genetic loci distributed around the chromosome and indexes variation that is subject to stabilising selection. It appears to provide a level of discrimination comparable to the NG-MAST typing scheme [15], although this has not been formally validated as different datasets have been used. MLST, however, has the advantage that isolate information is available alongside genotypic data in an established, publicly accessible database [23,24]. Unlike schemes that rely on antigen gene variation [5,15,29], which is subject to diversifying immune selection, MLST data can also be used to examine the evolutionary relationships among strains.

A total of 149 gonococcal isolates were typed by MLST in the present study. While only 27 polymorphisms were detected among the seven loci, a total of 66 unique allele combinations, or STs, were recorded. The low level of nucleotide diversity among gonococci inevitably results in a tighter clustering of these isolates in phylogenetic trees compared to meningococcal and *N. lactamica *isolates when concatenated sequences are analysed. However, the use of allelic profiles demonstrates a comparable level of discriminatory power to MLST of *N. meningitidis *and *N. lactamica*. The gonococcal STs were well differentiated with some showing temporal and geographic persistence. For instance, isolates with ST-1596 and ST-1583 were first isolated in Liverpool during the 1980s and have since been isolated in 2000, suggesting they may have a fitness advantage that has enabled them to persist in the population for over a decade. A total of 31 STs were represented by more than one isolate, with one group, ST-1579 represented by 10 isolates from three different countries, suggesting that isolates were distributed widely and not structured geographically. No temporal structuring was evident either, as the isolates collected in Liverpool between 1981–1989 were not significantly different from those collected in the same location between 2000 and 2001 (*p *> 0.05). While there was no evidence of geographical or temporal structuring in the gonococcal populations, UK isolates predominated in this study and some were from undefined locations, which may have influenced the outcome of the analysis. Structuring may be evident if more geographically and temporally diverse isolate collections were examined by MLST.

MLST provides a useful tool to study both the local and global distribution of isolates such as those with the AHU- phenotype, making it possible to track particular variants and examine transmission patterns. Of the 13 AHU- gonococci in the present study, 12 had identical genotypes with one differing by a single synonymous mutation at one locus. This illustrates the close relationship of this group and the ability of MLST to differentiate isolates with this auxotype. Further validation of the method would be required before MLST was used to resolve questions related to an outbreak situation and it may be necessary to complement it with antigen gene sequencing, as used in meningococcal epidemiology [30].

As the gonococcal MLST scheme uses nucleotide sequence data from exactly the same gene fragments as the meningococcal scheme, it can be used to compare MLST data from different *Neisseria *species, allowing phylogenetic and population genetic inferences to be made. The gonococcal MLST data were compared to data from studies of *N. meningitidis *and *N. lactamica *isolate collections previously published by the authors [16,20,25]. The use of these data, as opposed to the entire *Neisseria *MLST database was preferred as they had been extensively characterized and their provenance could be confirmed.

Like the *N. lactamica *and *N. meningitidis *alleles, the *d*_*N*_*/d*_*S *_ratios of the gonococcal alleles suggest that these loci evolve slowly and are not affected by diversifying selection, making them suitable for analysing evolutionary relationships among these species. However, this ratio could be affected by the small number of polymorphisms present within the collection. The MLST data for the three individual species were examined using: (1) the allelic profiles, (2) the individual alleles at each locus, and (3) the concatenated sequences for each allelic profile. When the MLST profiles were compared, the STs were unique to each species. The allele sequences were also species specific and no alleles were common among the neisseriae when 149 gonococcal, 324 meningococcal, and 103 *N. lactamica *isolates were examined. When additional *N. lactamica *isolates from the carriage study [20] and from German and Czech collections (unpublished data, not shown) were included in the analysis, only two alleles were common to more than one species. These alleles, at the *pgm *locus, were present among both *N. gonorrhoeae *and *N. lactamica *isolates. Alleles at this locus are among the least variable in gonococci. Thus, it seems more probable that these common alleles are a result of shared ancestry rather than interspecies recombination.

One of the advantages of a common MLST scheme for the neisseriae is that it can be used to distinguish between the *Neisseria *species and to identify unknown or misclassified isolates. Both neighbour-joining and split decomposition methods, using the concatenated MLST data, clustered the isolates into three distinct groups. The clustering of the STs into groups suggests that minimal recombination occurs among the housekeeping genes of these three *Neisseria*. This is confirmed by the *F*_*ST *_analysis, which suggests low levels of recombination among the species, the high number of fixed differences, the low number of shared polymorphisms, and the lack of alleles shared among species. Interestingly, the split decomposition analysis clustered the gonococcal sequences within the diversity of the meningococcus, reflecting the close ancestral relationship between these bacteria [31].

Although genetic recombination has been reported among *N. gonorrhoeae*, *N. lactamica *and *N. meningitidis *[32,33], the physical and temporal separation of these species within the human host is likely to contribute to a low frequency of interspecies recombination. *N. gonorrhoeae*, which colonises the urogenital tract, is rarely found in children as it is sexually transmitted and is only occasionally found in adult throats; *N. meningitidis *is carried in the throats of approximately 10% of the adult population [34] but is rarely carried by young children and found infrequently in the urogenital tract; *N. lactamica *is carried by only about 2% of adults [35] but is highly prevalent in young children with carriage rates of around 40 % [36,37]. While this limits opportunity for interspecies recombination, it does not affect intraspecies recombination, which may occur frequently creating an increasing number of STs from the available pool of alleles for each species, as has been observed in meningococci [25].

The results of the present study are inconsistent with a previous report that the *Neisseria *housekeeping alleles used in MLST were widely distributed among the neisseriae due to frequent interspecies recombination [38]. This was an *in silico *study that compared sets of 500 meningococcal STs downloaded from the *Neisseria *MLST database with all STs assigned to other named *Neisseria *species in the same database. The data that were analysed were not verified experimentally and access to the original samples was not requested. The present study did not include any of the apparently hybrid STs as they did not form part of the coherent populations analysed. We investigated these apparent hybrid STs present in the database for which samples were available. In all cases these were STs generated from historical freeze-dried cultures from which it was impossible to grow live organisms. Further analysis of the DNA samples suggested these were from mixed cultures and that the hybrid STs were a consequence of differential amplification of some loci. In conclusion, the present study finds little experimental support for extensive interspecies recombination among housekeeping genes in the *Neisseria*.

The lack of congruence among all of the phylogenetic trees may be a consequence of either shared ancestry or infrequent genetic exchange among the species. The relatively short lengths of the individual sequences used would also reduce any phylogenetic signal and therefore concatenated sequences were used to improve resolution. Only the tree for the alleles at the *gdh *locus produced a tree congruent with that obtained from the concatenated nucleotide sequences. The *gdh *locus may have evolved more rapidly than the other loci as these species diverged away from the ancestral population, creating *gdh *alleles that appear highly distinct for each species as shown in the *gdh *gene tree.

The species specificity of the *gdh *alleles and the congruence of the *gdh *gene tree with that produced from the concatenated sequences suggest that analysing sequences at this locus alone may be useful in differentiating among these three species and might help identify misclassified isolates. Occasionally *Neisseria *are misidentified [39,40], therefore a typing tool that can be exploited to differentiate species using either MLST profiles, allele sequences at particular loci or the concatenated gene sequences, could prove extremely helpful alongside traditional microbiological methods. This is especially important if commensal species are misidentified as *N. gonorrhoeae*, which could lead to serious social, legal and medical consequences [41].

Although a number of other commensal neisseriae have been typed (unpublished results from the MLST database [23,24]), these were not included in this study as too few isolates of these species have been typed for robust, meaningful analyses. MLST of representative collections of these other commensals, in particular *Neisseria polysaccharea *and *Neisseria cinerea*, which are closely related to the pathogenic *Neisseria *[42] would be advantageous, as knowledge of the genotypes of these species could be applied to species definitions and could facilitate identification of misclassified isolates.

## Conclusion

This analysis has shown that MLST can be used effectively to characterise *N. gonorrhoeae *collections, obtained both locally and globally, and has demonstrated a level of discrimination that appears comparable to that determined for the meningococcus using MLST [16,25] and the gonococcus using the NG-MAST scheme [15]. As an identical scheme has been used to characterize both *N. meningitidis *and *N. lactamica*, these data can be exploited to help define the three species, using either STs, individual alleles, in particular those at the *gdh *locus, or by concatenating the MLST data.

## Methods

### Bacterial isolates

A total of 149 gonococcal DNA samples were analysed, including 58 from Liverpool, collected between 1981 and 1991 and 38 from Liverpool collected between 2000 and 2001, one of which was known to be AHU-. A collection of 33 samples were obtained from isolates provided by the Genitourinary Infections Reference Laboratory, Gonococcus Reference Unit, Public Health Laboratory, Bristol, UK, and consisted of isolates that were geographically and epidemiologically unlinked. These included 12 isolates from cases of uncomplicated gonorrhoea from the UK (Colchester, London-Central Middlesex, South Wales, Epsom, Bristol, East London, Tyneside, Greenwich, Nottingham, Haverfordwest, and Birmingham), 11 isolates from cases of uncomplicated gonorrhoea from elsewhere in the world (Thailand, USA, South Africa, Pakistan, Uzbekistan, Hong Kong, Ireland, Bangladesh, Russia, Taiwan, and Spain), and 10 isolates from different and unrelated cases of DGI (five blood cultures and five joint fluid isolates). The remaining DNA samples were obtained from five isolates collected in Africa (two from Malawi and three from Nigeria), a collection of 12 AHU- isolates, and three reference isolates (FA19, FA1090, F62). Information regarding these isolates is available from the *Neisseria *MLST database [23,24].

### DNA preparation

DNA was extracted from 100 *μ*l of boiled cell suspensions obtained from gonococci collected in Liverpool with the Isoquick nucleic acid extraction kit (ISC Bioexpress, Kaysville, UT, USA), used in accordance with the manufacturer's instructions. Samples collected elsewhere were provided as pure chromosomal DNA.

### MLST

PCR amplifications and sequencing of the seven *Neisseria *MLST housekeeping gene fragments: *abcZ*, *adk*, *aroE, fumC, gdh*, *pdhC*, and *pgm *were undertaken with the oligonucleotide primers detailed in Table 4 using the protocol described previously [43]. All nucleotide sequences were determined directly from the PCR products. Briefly, sequence templates were generated using the PCR, and purified by precipitation with polyethylene glycol and sodium chloride [44]. The termination products were generated by cycle sequencing with the appropriate primers and BigDye terminators (Applied Biosystems). The products were then separated with an ABI prism 3700 automated DNA analyser. The sequence of each strand was determined at least once, and the resultant DNA sequences were assembled using the STADEN suite of computer programs [45]. Allele numbers and sequence types (STs) were assigned by querying the *Neisseria *MLST database [23,24].

**Table 4:** Oligonucleotide primers used in the *N. gonorrhoeae *MLST scheme

Locus	Name	Sequence (5'-3')	Function
*abcZ*	abcZ-P1	AATCGTTTATGTACCGCAGG	Amplification and sequencing
*abcZ*	abcZ-S2	GAGAACGAGCCGGGATAGGA	Amplification and sequencing
*ad*k	adk-P1	ATGGCAGTTTGTGCAGTTGG	Amplification
*ad*k	adk-P2	GATTTAAACAGCGATTGCCC	Amplification
*ad*k	adk-S1	AGGCTGGCACGCCCTTGG	Sequencing
*ad*k	adk-S2	CAATACTTCGGCTTTCACGG	Sequencing
*aroE*	aroE-P1	ACGCATTTGCGCCGACATC	Amplification and sequencing
*aroE*	aroE-P2	ATCAGGGCTTTTTTCAGGTT	Amplification
*aroE*	aroE-S2	ATGATGTTGCCGTACACATA	Sequencing
*fumC*	fumC-P1	CACCGAACACGACACGATGG	Amplification
*fumC*	fumC-P2	ACGACCAGTTCGTCAAACTC	Amplification
*fumC*	fumC-S1	TCCGGCTTGCCGTTTGTCAG	Sequencing
*fumC*	fumC-S2	TTGTAGGCGGTTTTGGCGAC	Sequencing
*gdh*	gdh-P1	ATCAATACCGATGTGGCGCGT	Amplification
*gdh*	gdh-P2	GGTTTTCATCTGCGTATAGAG	Amplification and sequencing
*gdh*	gdh-S3	CCTTGGCAAAGAAAGCCTGC	Sequencing
*pdhC*	pdhC-P1	GGTTTCCAACGTATCGGCGAC	Amplification
*pdhC*	pdhC-P2	ATCGGCTTTGATGCCGTATTT	Amplification and sequencing
*pdhC*	pdhC-S1	TCTACTACATCACCCTGATG	Sequencing
*pgm*	pgm-S1	CGGCGATGCCGACCGCTTGG	Amplification and sequencing
*pgm*	pgm-S2	GGTGATGATTTCGGTTGCGCC	Amplification and sequencing

Oligonucleotide primers were as previously published [16,25].

### Data analysis

The computer program START, version 1.05 [46] was utilised to examine the number of polymorphic sites and the ratios of nonsynonymous to synonymous nucleotide substitutions (*d*_*N*_*/d*_*S*_) among the alleles. Nucleotide sequences from the seven loci were concatenated in-frame to produce single sequences of length 3282 bp for each ST, using the concatenation tool found at the PubMLST website [23,24]. DnaSP, version 4 [47], was used to calculate shared polymorphisms and fixed differences [48] between the isolate collections, and *F*_*ST *_values [49] were calculated using Arlequin, version 2, [50]. The *F*_*ST *_statistic measures the extent of genetic differentiation and computes an average level of gene flow, so that an *F*_*ST *_value of zero would indicate free genetic recombination, whereas an *F*_*ST *_*value *of one would indicate that recombination is unlikely). Neighbour-joining trees were drawn from the concatenated MLST alleles and the individual allele sequences using MEGA, version 2.1 [51], which was also used to measure nucleotide sequence divergence. All three coding positions were examined and the Kimura 2-parameter distance correction [52] was applied. The concatenated sequence data were also visualised using split decomposition analysis, using hamming distances with SplitsTree, version 3.1 [53]. The reliability of the inferred phylogenies was evaluated using bootstrap tests (2000 replications).

## Authors' contributions

JSB designed the study, undertook MLST of the *N. gonorrhoeae *isolates, analysed and interpreted the results, and drafted the manuscript. KAJ reviewed the manuscript and maintains the *Neisseria *MLST website and database. MCJM conceived of the study and contributed to the drafting of the manuscript. IMF contributed to the drafting of the manuscript. CAH, NJS and PFS provided DNA samples from gonococcal isolate collections and provided information relating to these isolates, where available.

## Supplementary Material

Additional file 1 The 149 gonococcal isolates used in this study in MS Word format Click here for file
