# Supplementary material for: Species status of Neisseria gonorrhoeae: evolutionary and epidemiological inferences from multilocus sequence typing
Source: BMC Biol. 2007 Sep 7;5:35. doi: 10.1186/1741-7007-5-35 (PMC2031879; doi:10.1186/1741-7007-5-35)
Supplement: Additional file 1 — The 149 gonococcal isolates used in this study in MS Word format [file 1741-7007-5-35-S1.doc]

### Supplementary Table - The 149 gonococcal isolates used in this study

| Isolate | ST | Country | Region | Date | Provider |
| --- | --- | --- | --- | --- | --- |
| 22584 | 1579 | USA | ? | ? | PHL, Bristol, UK |
| 27706 | 1579 | UK | S. Wales | ? | PHL, Bristol, UK |
| 27728 | 1579 | UK | Tyneside | ? | PHL, Bristol, UK |
| 28252 | 1579 | UK | Haverford-West | ? | PHL, Bristol, UK |
| 28480 | 1579 | UK | London/central Middlesex | ? | PHL, Bristol, UK |
| 28516 | 1579 | UK | Colchester | ? | PHL, Bristol, UK |
| F1167 | 1579 | UK | Liverpool | 22/06/2000 | C. Anthony Hart |
| F2221 | 1579 | UK | Liverpool | 19/07/2000 | C. Anthony Hart |
| M2020 | 1579 | UK | Liverpool | 10/07/2000 | C. Anthony Hart |
| M2549 | 1579 | UK | Liverpool | 25/08/2000 | C. Anthony Hart |
| F2570 | 1580 | UK | Liverpool | 23/08/2000 | C. Anthony Hart |
| M1986 | 1580 | UK | Liverpool | 04/07/2000 | C. Anthony Hart |
| M2002 | 1580 | UK | Liverpool | 06/07/2000 | C. Anthony Hart |
| M2178 | 1580 | UK | Liverpool | 24/07/2000 | C. Anthony Hart |
| M940063 | 1580 | UK | Liverpool | 07/06/2000 | C. Anthony Hart |
| F2224 | 1581 | UK | Liverpool | 19/07/2000 | C. Anthony Hart |
| M1658 | 1581 | UK | Liverpool | 06/06/2000 | C. Anthony Hart |
| M2465 | 1581 | UK | Liverpool | 18/08/2000 | C. Anthony Hart |
| GC12 | 1582 | UK | Liverpool | 2000/2001 | C. Anthony Hart |
| GC23 | 1582 | UK | Liverpool | 2000/2001 | C. Anthony Hart |
| GC27 | 1582 | UK | Liverpool | 2000/2001 | C. Anthony Hart |
| GC4 | 1583 | UK | Liverpool | 2000/2001 | C. Anthony Hart |
| M2553 | 1583 | UK | Liverpool | 29/08/2000 | C. Anthony Hart |
| F82/10 | 1583 | UK | Liverpool | 1982 | C. Anthony Hart |
| F82/253 | 1583 | UK | Liverpool | 1982 | C. Anthony Hart |
| M2336 | 1584 | UK | Liverpool | 08/08/2000 | C. Anthony Hart |
| M973122 | 1584 | UK | Liverpool | 03/07/2000 | C. Anthony Hart |
| 26593 | 1585 | ? | ? | ? | PHL, Bristol, UK |
| 29528 | 1585 | UK | Greenwich | ? | PHL, Bristol, UK |
| F1722 | 1585 | UK | Liverpool | 12/06/2000 | C. Anthony Hart |
| M2466 | 1585 | UK | Liverpool | 18/08/2000 | C. Anthony Hart |
| GC1 | 1586 | UK | Liverpool | 2000/2001 | C. Anthony Hart |
| M2236 | 1586 | UK | Liverpool | 27/07/2000 | C. Anthony Hart |
| 28837 | 1587 | Spain | ? | ? | PHL, Bristol, UK |
| GC21 | 1587 | UK | Liverpool | 2000/2001 | C. Anthony Hart |
| GC30 | 1587 | UK | Liverpool | 2000/2001 | C. Anthony Hart |
| GC26 | 1588 | UK | Liverpool | 2000/2001 | C. Anthony Hart |
| GC40 | 1588 | UK | Liverpool | 2000/2001 | C. Anthony Hart |
| F981 | 1589 | Nigeria | ? | 05/06/2000 | C. Anthony Hart |
| F982 | 1589 | Nigeria | ? | 05/06/2000 | C. Anthony Hart |
| 28897 | 1590 | Taiwan | ? | ? | PHL, Bristol, UK |
| 28622 | 1590 | UK | E. London | ? | PHL, Bristol, UK |
| M1959 | 1590 | UK | Liverpool | 03/07/2000 | C. Anthony Hart |
| M2183 | 1591 | UK | Liverpool | 24/07/2000 | C. Anthony Hart |
| M1651 | 1592 | UK | Liverpool | 05/06/2000 | C. Anthony Hart |
| M1635 | 1593 | UK | Liverpool | 05/06/2000 | C. Anthony Hart |
| 28839 | 1594 | UK | Bristol | ? | PHL, Bristol, UK |
| 28962 | 1594 | Ireland | ? | ? | PHL, Bristol, UK |
| M1743 | 1594 | UK | Liverpool | 14/06/2000 | C. Anthony Hart |
| F971055 | 1595 | UK | Liverpool | 13/07/2000 | C. Anthony Hart |
| FA1012 | 1595 | ? | ? | ? | P. Frederick Sparling |
| FA1031 | 1595 | ? | ? | ? | P. Frederick Sparling |
| FA1033 | 1595 | ? | ? | ? | P. Frederick Sparling |
| FA1047 | 1595 | ? | ? | ? | P. Frederick Sparling |
| FA1080 | 1595 | ? | ? | ? | P. Frederick Sparling |
| FA1085 | 1595 | ? | ? | ? | P. Frederick Sparling |
| FA1092 | 1595 | ? | ? | ? | P. Frederick Sparling |
| FA1095 | 1595 | ? | ? | ? | P. Frederick Sparling |
| FA3002 | 1595 | ? | ? | ? | P. Frederick Sparling |
| FA7035 | 1595 | ? | ? | ? | P. Frederick Sparling |
| FA1089 | 1595 | ? | ? | ? | P. Frederick Sparling |
| 28539 | 1596 | ? | ? | ? | PHL, Bristol, UK |
| F1677 | 1596 | UK | Liverpool | 01/06/2000 | C. Anthony Hart |
| BE/3292 | 1596 | UK | Liverpool | 1987 | C. Anthony Hart |
| M91/987 | 1596 | UK | Liverpool | 1991 | C. Anthony Hart |
| GC14 | 1597 | UK | Liverpool | 2000/2001 | C. Anthony Hart |
| GC15 | 1598 | UK | Liverpool | 2000/2001 | C. Anthony Hart |
| GC29 | 1599 | UK | Liverpool | 2000/2001 | C. Anthony Hart |
| GC31 | 1600 | UK | Liverpool | 2000/2001 | C. Anthony Hart |
| C774 | 1601 | Malawi | ? | 20/07/1987 | C. Anthony Hart |
| AZ/240 | 1601 | UK | Liverpool | 1983 | C. Anthony Hart |
| BC/2096 | 1601 | UK | Liverpool | 1985 | C. Anthony Hart |
| D779 | 1602 | Malawi | ? | 10/06/1991 | C. Anthony Hart |
| F980 | 1603 | Nigeria | ? | 05/06/2000 | C. Anthony Hart |
| 25563 | 1889 | ? | ? | ? | PHL, Bristol, UK |
| 27833 | 1890 | Pakistan | ? | ? | PHL, Bristol, UK |
| 26399 | 1891 | ? | ? | ? | PHL, Bristol, UK |
| 27806 | 1892 | UK | Birmingham | ? | PHL, Bristol, UK |
| FA19 | 1892 | ? | ? | ? | Lab strain, Nigel J. Saunders |
| 25527 | 1893 | ? | ? | ? | PHL, Bristol, UK |
| 25562 | 1893 | ? | ? | ? | PHL, Bristol, UK |
| 26775 | 1894 | ? | ? | ? | PHL, Bristol, UK |
| 29214 | 1895 | Hong Kong | ? | ? | PHL, Bristol, UK |
| 25448 | 1896 | UK | Bristol | ? | PHL, Bristol, UK |
| 28386 | 1897 | UK | Nottingham | ? | PHL, Bristol, UK |
| 25534 | 1898 | ? | ? | ? | PHL, Bristol, UK |
| FA1090 | 1899 | ? | ? | ? | Lab strain, Nigel J. Saunders |
| F62 | 1900 | ? | ? | ? | Lab strain, Nigel J. Saunders |
| 27361 | 1901 | UK | Epsom | ? | PHL, Bristol, UK |
| 22159 | 1902 | South Africa | ? | ? | PHL, Bristol, UK |
| F87/1856 | 1902 | UK | Liverpool | 1987 | C. Anthony Hart |
| 27886 | 1903 | Bangladesh | ? | ? | PHL, Bristol, UK |
| 23372 | 1904 | Thailand | ? | ? | PHL, Bristol, UK |
| 27921 | 1905 | Uzbekistan | ? | ? | PHL, Bristol, UK |
| 28197 | 1905 | Russia | ? | ? | PHL, Bristol, UK |
| 26034 | 1906 | ? | ? | ? | PHL, Bristol, UK |
| 26241 | 1906 | ? | ? | ? | PHL, Bristol, UK |
| F82/2262 | 1917 | UK | Liverpool | 1982 | C. Anthony Hart |
| F82/2269 | 1918 | UK | Liverpool | 1982 | C. Anthony Hart |
| AX/1484 | 1919 | UK | Liverpool | 1981 | C. Anthony Hart |
| F82/1518 | 1919 | UK | Liverpool | 1982 | C. Anthony Hart |
| F82/1710 | 1919 | UK | Liverpool | 1982 | C. Anthony Hart |
| F82/1771 | 1919 | UK | Liverpool | 1982 | C. Anthony Hart |
| F82/21771 | 1919 | UK | Liverpool | 1982 | C. Anthony Hart |
| BG/1348 | 1920 | UK | Liverpool | 1989 | C. Anthony Hart |
| BG/1921 β-lactamase neg | 1920 | UK | Liverpool | 1989 | C. Anthony Hart |
| BG/1921 β-lactamase pos | 1920 | UK | Liverpool | 1989 | C. Anthony Hart |
| BG/2067 | 1920 | UK | Liverpool | 1989 | C. Anthony Hart |
| BG/2246 | 1920 | UK | Liverpool | 1989 | C. Anthony Hart |
| F89/2787 | 1920 | UK | Liverpool | 1989 | C. Anthony Hart |
| F82/1075 | 1921 | UK | Liverpool | 1982 | C. Anthony Hart |
| F82/1739 | 1922 | UK | Liverpool | 1982 | C. Anthony Hart |
| BD/1365A | 1923 | UK | Liverpool | 1986 | C. Anthony Hart |
| F86/1430 | 1923 | UK | Liverpool | 1986 | C. Anthony Hart |
| F86/1703 | 1923 | UK | Liverpool | 1986 | C. Anthony Hart |
| F86/1736 | 1923 | UK | Liverpool | 1986 | C. Anthony Hart |
| BD/3091 | 1924 | UK | Liverpool | 1986 | C. Anthony Hart |
| F86/1674 | 1924 | UK | Liverpool | 1986 | C. Anthony Hart |
| F81/1546 | 1925 | UK | Liverpool | 1981 | C. Anthony Hart |
| AY/1179 | 1926 | UK | Liverpool | 1982 | C. Anthony Hart |
| F86/1366 | 1926 | UK | Liverpool | 1986 | C. Anthony Hart |
| BD/375 | 1927 | UK | Liverpool | 1986 | C. Anthony Hart |
| AX/1528 | 1928 | UK | Liverpool | 1981 | C. Anthony Hart |
| BE/740 | 1929 | UK | Liverpool | 1987 | C. Anthony Hart |
| F87/833 | 1929 | UK | Liverpool | 1987 | C. Anthony Hart |
| F87/956 | 1929 | UK | Liverpool | 1987 | C. Anthony Hart |
| BE/1229 | 1930 | UK | Liverpool | 1987 | C. Anthony Hart |
| BE/1263 | 1930 | UK | Liverpool | 1987 | C. Anthony Hart |
| BE/445 | 1930 | UK | Liverpool | 1987 | C. Anthony Hart |
| BE/674 | 1930 | UK | Liverpool | 1987 | C. Anthony Hart |
| F87/1449 | 1930 | UK | Liverpool | 1987 | C. Anthony Hart |
| AX/1729 | 1931 | UK | Liverpool | 1981 | C. Anthony Hart |
| AX/1879 | 1931 | UK | Liverpool | 1981 | C. Anthony Hart |
| F81/2139 | 1931 | UK | Liverpool | 1981 | C. Anthony Hart |
| F87/1261 | 1931 | UK | Liverpool | 1987 | C. Anthony Hart |
| AX/1894 | 1931 | UK | Liverpool | 1981 | C. Anthony Hart |
| AY/1683 | 1932 | UK | Liverpool | 1982 | C. Anthony Hart |
| F81/1501 | 1932 | UK | Liverpool | 1981 | C. Anthony Hart |
| F81/1911 | 1932 | UK | Liverpool | 1981 | C. Anthony Hart |
| F81/2217 | 1932 | UK | Liverpool | 1981 | C. Anthony Hart |
| F82/1333 | 1932 | UK | Liverpool | 1982 | C. Anthony Hart |
| BF/1074 | 1933 | UK | Liverpool | 1988 | C. Anthony Hart |
| F88/1569 | 1933 | UK | Liverpool | 1988 | C. Anthony Hart |
| F81/1500 | 1934 | UK | Liverpool | 1981 | C. Anthony Hart |
| AX/1855 | 1961 | UK | Liverpool | 1981 | C. Anthony Hart |
| AX/591 | 1962 | UK | Liverpool | 1981 | C. Anthony Hart |
| F87/1720 | 1963 | UK | Liverpool | 1987 | C. Anthony Hart |
| BE/2106 | 1964 | UK | Liverpool | 1987 | C. Anthony Hart |
| FA3000 | 5688 | ? | ? | ? | P. Frederick Sparling |
